# Research turns hope into reality

**DOI:** 10.7554/eLife.106706

**Published:** 2025-03-26

**Authors:** Anna C Greene, Casey S Greene

**Affiliations:** 1 Neuroendocrine Tumor Research Foundation Boston United States; 2 University of Colorado School of Medicine, Anschutz Medical Campus Aurora United States

**Keywords:** Science Under Threat in the United States, science, politics, science funding, cancer treatment, National Institutes of Health, healthcare provision, None

## Abstract

Two scientists describe how an acute myeloid leukemia diagnosis underscores the need for continued federal support for research and access to care.

Last September, during Labor Day weekend, our lives were upended when one of us, Anna, was diagnosed with acute myeloid leukemia after a routine physical. If you look up survival rates for acute myeloid leukemia, you see how devastating such a diagnosis this can be. Our world shattered. Would we both see our daughter, who had started first grade two weeks before, graduate from first grade? Elementary school? High school?

We trained as scientists and have worked in academic, industry, and non-profit settings. Over the past six years, Anna has worked in cancer-focused non-profits, funding research and supporting patients and families. The work has been a deeply meaningful opportunity to make a difference for people in life’s most challenging times that we never expected would become this personal.

Because we understood the power of research put into practice, we sought treatment at an academic medical center. We knew that being treated by physicians and physician-scientists on the cutting edge of leukemia research and treatment gave us the best hope. We quickly learned that acute myeloid leukemia is a complex collection of subtypes, each with its own treatment path and prognosis. Personalizing treatment based on subtypes is only possible because of decades of federally funded research and rigorous scientific inquiry. A targeted regimen offered the best shot at reducing side effects and increasing the odds of survival.

Our ability to access that treatment strategy underscores two critical issues we face in the United States: whether the treatments we need exist, and if we can access them. Both are under threat. The cancellation of grants or abrupt changes to funding policies that may sound esoteric to the public – such as significant changes to indirect cost rates for research grants – can derail life-saving studies. These funds keep the lights on in labs and pay for the facilities where breakthroughs happen, and cutting them will harm research, particularly at institutions without large endowments. At the same time, potential changes to Medicaid put access to crucial therapies, nearly all of which have been developed through public investment, at risk for many Americans.

As scientists, we must show how federal funding makes tangible differences in lives. For acute myeloid leukemia, decades of research have led to drugs targeting specific genetic mutations, drastically improving outcomes for some subtypes. The treatment strategies today are far more advanced than those of a generation ago. If funding cuts erode this momentum, we risk halting innovative research. Do we really want our children to grow up in a world where the care options available to them and their families are no better than those available today?

It is also critically important that we ensure that these breakthroughs reach Americans wherever they receive their care. Hospitals in rural communities are closing, and it is a struggle to maintain essential services, much less state-of-the-art care, in many areas. We need to recognize our role in advocating for access to care across the country. Increasingly, it appears that changes to Medicaid may make it harder for patients to access treatments and health systems to provide state-of-the-art care.

As scientists and citizens, now is a time to share stories with our neighbors, legislators, and communities that demonstrate research isn’t an ivory-tower pursuit; it is a lifeline for members of our communities. We need policymakers to ask how proposed changes impact these vital efforts. For scientists, advocating outside the lab can feel unnatural, but speaking up is a key step in safeguarding the breakthroughs our communities depend on.

Our story is not unique. Many families face life-changing diagnoses. For each one, the unifying questions are: what is the best available care, and is it within reach? As scientists, we are fundamentally in the business of hope – hope that our work delivers new knowledge that leads to better treatments, better outcomes, and better tomorrows. Research is how we turn hope into reality, and robust support for healthcare access ensures it reaches all who need it. When the unthinkable happens, every family should have hope in treatments developed through federal investment and faith in their ability to access them. While we work to protect the pipeline of new life-saving treatments, let us also ensure those treatments remain available to all. We must preserve and strengthen that lifeline because none of us knows when we – or someone we love – will need it.

## Note

AI-based tools were used to critique drafts of this article and to identify opportunities to better communicate with a broad audience.

